# An age–size reaction norm yields insight into environmental interactions affecting life-history traits: a factorial study of larval development in the malaria mosquito *Anopheles gambiae* sensu stricto

**DOI:** 10.1002/ece3.589

**Published:** 2013-05-18

**Authors:** Conan Phelan, Bernard D Rotiberg

**Affiliations:** 1 Division of Biological Sciences, University of MontanaMissoula, Canada; 2 Department of Biological Sciences, Simon Fraser UniversityBurnaby, Canada

**Keywords:** Age, factor, food, growth, insect, interaction effect, size, temperature, vector, water depth

## Abstract

Environmental factors frequently act nonindependently to determine growth and development of insects. Because age and size at maturity strongly influence population dynamics, interaction effects among environmental variables complicate the task of predicting dynamics of insect populations under novel conditions. We reared larvae of the African malaria mosquito *Anopheles gambiae* sensu stricto (s.s.) under three factors relevant to changes in climate and land use: food level, water depth, and temperature. Each factor was held at two levels in a fully crossed design, for eight experimental treatments. Larval survival, larval development time, and adult size (wing length) were measured to indicate the importance of interaction effects upon population-level processes. For age and size at emergence, but not survival, significant interaction effects were detected for all three factors, in addition to sex. Some of these interaction effects can be understood as consequences of how the different factors influence energy usage in the context of a nonindependent relationship between age and size. Experimentally assessing interaction effects for all potential future sets of conditions is intractable. However, considering how different factors affect energy usage within the context of an insect's evolved developmental program can provide insight into the causes of complex environmental effects on populations.

## Introduction

**Figure 1:**
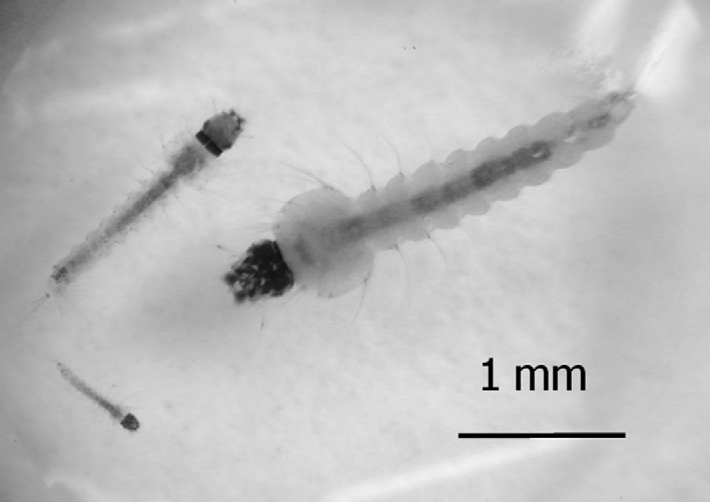
Various instars of larval *Anopheles gambiae* sensu stricto. This mosquito is a major vector of human malaria in Africa.

Any given environment is comprised of myriad factors, and these factors often interact with one another to affect growth and development of larval insects in nonlinear ways. Such effects have been demonstrated in several studies (Colbo and Porter 1981; Stillwell et al. 2007; Triggs and Knell 2011), and mosquitoes are no exception. Interactions among environmental factors have been shown to affect life-history traits in multiple mosquito species (Lyimo et al. 1992; Leonard and Juliano 1995; Juliano 1996; Walker et al. 1997a; Baltzley et al. 1999; Agnew et al. 2002; Knight et al. 2004). This includes a principal vector of human malaria in Africa, *Anopheles gambiae* sensu stricto (s.s.). (Levine et al. 2004; Fig. 1); its survival, development time, and body size have been shown to respond in nonlinear ways to combinations of larval rearing density and water temperature (Lyimo et al. 1992). Reliable climate-based predictions of mosquito dynamics and malaria have been evasive (Chaves and Koenraadt 2010). A better understanding of how conditions affect population dynamics of mosquitoes would improve predictive models of vector abundance and disease risk, which would further help managers and policy makers combat malaria (Parham et al. 2012; C. Beierkuhnlein, pers. comm.).

The general question of how environmental conditions affect malaria vectors has received substantial scientific attention. Many studies have measured associations between larval habitat characteristics and mosquito abundance (Gimnig et al. 2001; Minakawa et al. 2004; Impoinvil et al. 2008; Fillinger et al. 2009). But the correlative approach used in such studies is limited because future conditions frequently fall outside those under which statistical models are derived (Williams and Jackson 2007; Buckley et al. 2010). Toward a more nuanced understanding of environmental effects, several manipulative studies have tested how various factors affect growth and development of disease-transmitting mosquitoes (Briegel 2003). Such studies have focused on how the environment affects life-history traits such as larval survival, development time, body size, metabolic condition, and fecundity because these traits are linked to population dynamics (Roff 1992). And different environmental factors that affect life-history traits often produce interaction effects that appear idiosyncratic (Lyimo et al. 1992). A better understanding of what causes these interactions is needed.

An important first step toward improving understanding of complex environmental effects for insects is to better characterize such effects (Reiskind and Zarrabi 2012). In *An. gambiae* s.s., multiple factors are known to influence two important life-history traits, age and size at metamorphosis. Both of these traits affect how populations will change over time because each one strongly influences population growth; age at maturity affects generation time, whereas adult size determines fecundity (Roff 1992; Lyimo and Takken 1993). These traits, age and size at metamorphosis, are both affected by three factors that are relevant to climate change and land use. These are food availability, water depth, and water temperature. The general, or independent, effects of these factors upon age and size, as well as larval survival, are already known for this species. More food tends to result in shorter time to metamorphosis, greater adult size, and greater larval survival (Timmermann and Briegel 1996; Gimnig et al. 2002). The effects of deeper water are the inverse to that of more food. The optimal water depth for *An. gambiae* s.s. larvae is quite shallow. In a lab study, Timmermann and Briegel (1993) found it to be less than 2 cm, and as depth increased beyond this level larval survival and adult size both decreased. For the broader species complex *An. gambiae* sensu lato (of which sensu stricto is a member), Ndenga et al. (2011) found 11–900 times greater incidence of larvae in shallow puddles (average depth 7 cm) over other habitats. The viable temperature range for development of *An. gambiae* s.s. has been shown to be 18–32°C, with minimal mortality at around 24°C (Bayoh and Lindsay 2004). Within the viable range, increasing water temperature yields both shorter developmental times and smaller size at emergence (Lyimo et al. 1992) (thus conforming to the widespread pattern called the temperature–size rule, whereby most ectotherms mature at a smaller size under higher temperatures [Angilletta and Sears 2004]). Each of these three factors, food, water depth, and temperature affects growth and development of *An. gambiae* s.s. within the context of this species' evolved life history. However, it is not clear how these factors mediate one another's effects.

Interdependent effects of multiple factors can be considered in the context of phenotypic plasticity. Phenotypically plastic traits change as a function of the environment. For instance, in *An. gambiae* s.s., age and size at emergence respond to food, temperature, and depth. The relationship between an environmental factor and the trait it influences is called a reaction norm. Reaction norms are typically displayed as graphs with the environmental gradient on the *x*-axis and the plastic trait values on the *y*-axis. If the shape of a reaction norm along one environmental axis is unaffected by other environmental axes (i.e., factors), the reaction norm is simple (Stillwell et al. 2007). In contrast, when there are interaction effects the reaction norm is complex and its shape or slope will depend on multiple variables.

A further source of complexity is that plastic traits can be nonindependent. During growth, size is a function of time, and consequently age and size at maturity are nonindependent. When plotted across a range of environmental qualities, animals that exhibit plastic age and size at metamorphosis often show an L-shaped relationship between age and size (Wilbur and Collins 1973; Plaistow et al. 2004). This pattern has been demonstrated in many animal systems, including *An. gambiae* s.s. and other mosquitoes (Carpenter 1984; Walker et al. 1997a; Gimnig et al. 2002; C. Phelan unpubl. data). Although it is clear that different factors influence growth and development, to our knowledge this age–size reaction norm has not been assessed across multiple factors at once. It remains unclear whether an L-shaped relationship between age and size remains constant across changes in more than one factor; is the age–size reaction norm simple or complex for *An. gambiae* s.s.? If simple, different rearing environments that result from changes in multiple factors would cause the age–size response to shift along a constant species-specific curve. If complex, interaction effects will be evident and the underlying position or shape of the curve will change under different factor levels.

We conducted a factorial rearing experiment in the lab to determine how food availability, water depth, and water temperature influenced age at emergence, size at emergence, and survival of larval *An. gambiae* s.s. Our goals were to (1) identify dependencies among factors (i.e., statistical interactions) and (2) evaluate whether the responses of *An. gambiae* s.s. fall out along a single L-shaped age–size reaction norm. Plastic development can be adaptive for dealing with environmental changes that are encountered during the larval period. It is also relevant to differences among discrete larval habitats that remain relatively constant through time. This factorial rearing experiment examined plastic responses of *An. gambiae* s.s. in this latter context of spatial, rather than temporal, variation in rearing conditions.

## Materials and Methods

We manipulated food level, water depth, and water temperature for individual *An. gambiae* s.s. larvae. Each factor was held at two levels (low and high) to give a 2 × 2 × 2 design. The levels for each factor were selected using three pilot experiments in which individual larvae were raised across a range of values for the focal factor while the other two were held constant at reasonable levels. The levels chosen for the factorial experiment showed substantial effects upon age and size at emergence while still being well within the upper and lower bounds of larval responses and yielding high survival (C. Phelan, unpublished data). In the factorial experiment we tracked larval survival during development, recorded days to emergence, and measured adult wing length as an index of size (Koella and Lyimo 1996). Larvae were reared individually in cylindrical Drosophila vials (diameter 25 mm, height 95 mm). Food levels were 0.2 or 1.4 mg/larva/day (Nutrafin basix staple for tropical fish, Hagen Inc., Quebec, Canada). Water depths in the rearing vials were maintained at either 2 or 5 cm. Water temperatures were maintained at either 26 or 30°C using a series of water baths. Constant temperatures were used because we lacked the ability to vary temperature diurnally and we wished to avoid any complications from comparing variable levels. Prior to addition of food each day, we used a pipette to remove accumulated food residue and waste material from the bottom of the vial to prevent fouling (M. J. Voordouw, pers. comm.).

Larvae hatched, on 28 November 2009, from eggs that originated from a 25 November blood feed. The newly hatched larvae were initially in a group culture and fed ground fish food ad libitum. They were transferred to vials on 29 November and the experimental treatments began. Each day, a 1-mL suspension of ground food was added to each vial. Food was administered by pipette, and a magnetic stirrer was used to keep a consistent mixture of food and water prior to transfer. Vial temperature was controlled by a system of eight water baths, four at each temperature. These baths were arranged such that the two temperatures alternated spatially. Vials were held in racks of eight and there were 20 racks for a total of 160 vials, with either 16 or 24 vials in each bath. Each rack of eight contained two full sets of randomly arranged food-by-depth treatments, and the number of vials at each temperature was equal. Individuals that survived to emerge as adults were freeze killed, sexed, and their wing lengths measured using a stage micrometer under a dissecting microscope. Wing length was measured as distance between the alula notch and the wing tip.

Statistical analyses were done using R version 2.10.1 for Linux (R-Development-Core-Team 2010). Survival was analyzed using a generalized linear model with a binomial error distribution (Quinn and Keough 2002; Crawley 2007). Two separate linear models were used to analyze age at emergence and wing length. Including water bath as a random factor did not improve the statistical models and so it was discarded (Pinheiro and Bates 2000). The generalized least-squares method and the varIdent function were used to address homoscedasticity in the data when necessary (Zuur et al. 2009; Table 1; Table 2). For the above models, we conducted backward selection using a critical α of 0.05 to achieve reduced models (Quinn and Keough 2002) while forcing the retention of main experimental factors. All two- and three-way interactions were tested and nonsignificant interactions were dropped. To account for interaction effects, adjusted means were calculated using the R effects package (Fox 2003). To explore the data in the context of the age–size reaction norm, we conducted a polynomial regression of days to emergence against wing length.

**Table 1:** Output from linear model of days to emergence for developing *Anopheles gambiae* sensu stricto

Source of variation	DF	*F*-value	*P*-value
Food	1	247.20	<0.0001
Depth	1	23.89	<0.0001
Temperature	1	61.25	<0.0001
Sex	1	6.57	0.0118
Food × depth	1	19.75	<0.0001
Food × sex	1	3.94	0.0498
Residuals	73		

Model terms are food availability, rearing temperature, water depth, and sex. Generalized least-squares method was used to address heteroscedasticity between food treatment levels (Zuur et al. 2009). Factors were selected using backward elimination until all *P*-values ≤ 0.05 or only main effects remained (Quinn and Keough 2002).

**Table 2:** Output of linear model of wing length for developing *Anopheles gambiae* sensu stricto

Source of variation	DF	*F*-value	*P*-value
Food	1	129.57	<0.0001
Depth	1	4.24	0.0421
Temperature	1	48.70	<0.0001
Sex	1	23.42	<0.0001
Food × depth	1	10.40	0.0017
Food × temperature	1	13.40	0.0004
Depth × temperature	1	6.18	0.0147
Depth × sex	1	18.75	<0.0001
Residuals	71		

Model terms are food availability, water depth, rearing temperature, and sex. A generalized least-squares model was used to address heteroscedasticity food treatment levels (Zuur et al. 2009). Factors were selected using backward elimination until all *P*-values ≤ 0.05 or only main effects remained (Quinn and Keough 2002).

## Results

**Figure 2:**
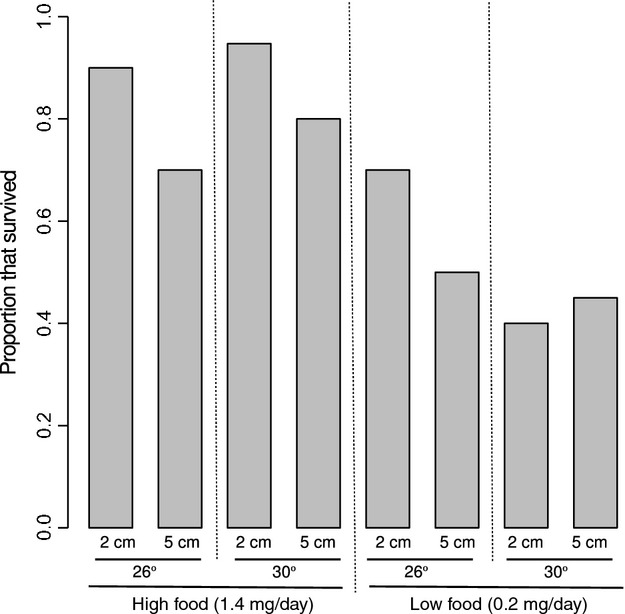
Proportion of *Anopheles gambiae* sensu stricto larvae that survived through emergence. Levels of each factor are indicated. Food was the only factor to significantly affect survival (*P* < 0.001).

The only experimental factor to significantly affect survival was food, with higher food yielding greater survival (*P* < 0.0001; Fig. 2).

**Figure 3:**
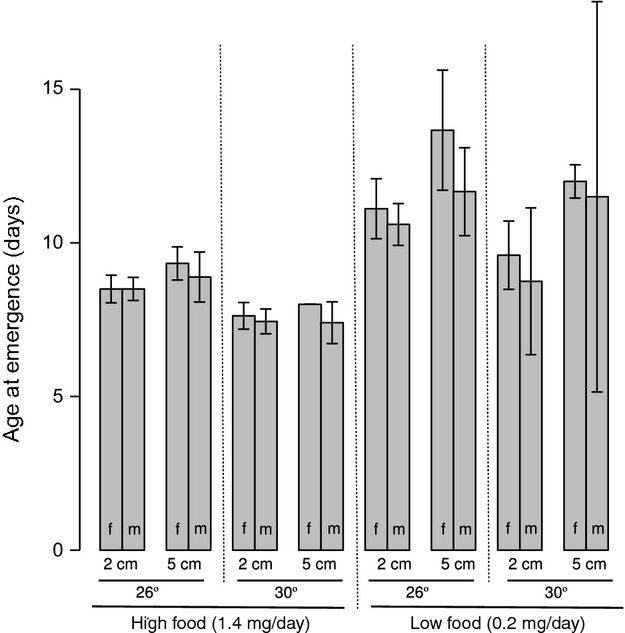
Mean age at emergence of *Anopheles gambiae* sensu stricto for each treatment and sex. Levels of each experimental factor as well as sex are indicated. Confidence intervals are 95%.

**Figure 4:**
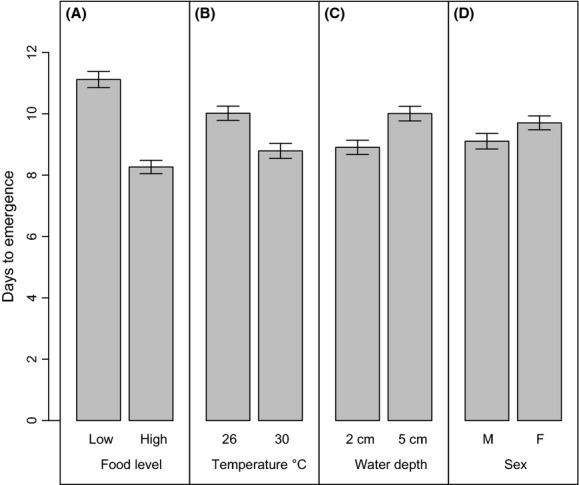
Adjusted means for age at emergence by factor and level. Panels A, B, C, and D show, respectively, the effects of food level, temperature, water depth, and sex. Adjusted means and 95% Confidence intervals were calculated using the effects package in R (Fox 2003).

**Figure 5:**
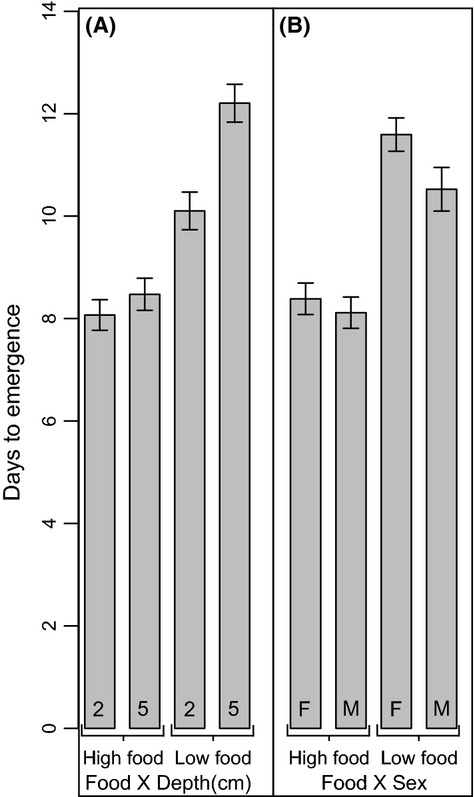
Adjusted mean age at emergence by groups showing interactions among factors. Panel A shows food-by-depth. Panel B shows food-by-sex. High- and low-food labels correspond to 0.2 and 1.4 mg per larva per day. Adjusted means and 95% Confidence intervals were calculated using the effects package in R (Fox 2003).

Age at emergence was affected by all three experimental factors, and by sex and the interactions food-by-depth and food-by-sex (Table 1). In general, higher food level decreased age at emergence (Figs. 3 and 4A), deeper water increased age at emergence (Figs. 3 and 4C), and higher temperature decreased age at emergence (Figs. 3 and 4B). Also, females generally took longer than males to develop (Figs. 3 and 4D). The food-by-depth interaction was such that deeper water depth caused a greater delay in emergence under low food than high (Fig. 5A). For the food-by-sex interaction, the difference between female and male development times was more pronounced under low food (Fig. 5B).

**Figure 6:**
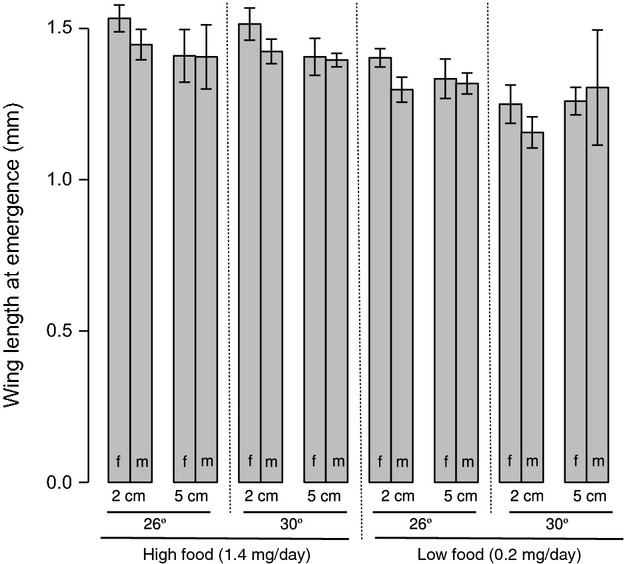
Mean adult wing length of *Anopheles gambiae* sensu stricto for each treatment and sex. Level of each experimental factor as well as sex is indicated. Confidence intervals are 95%.

**Figure 7:**
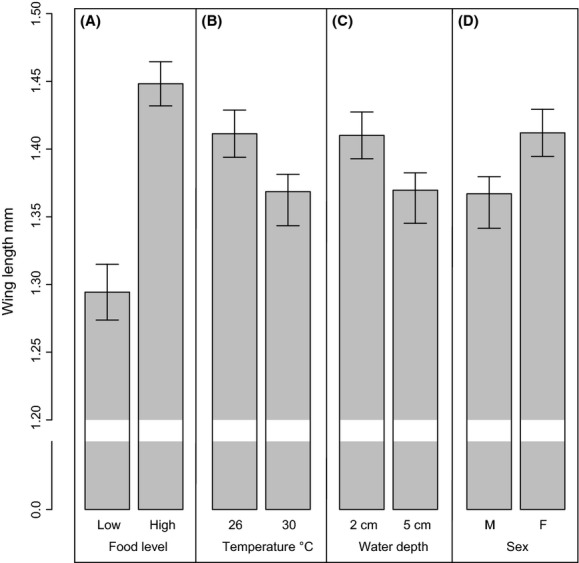
Adjusted mean wing lengths by factor and level. Panels A, B, C, and D show, respectively, the effects of food level, temperature, water depth, and sex. Adjusted means and 95% Confidence intervals were calculated using the effects package in R (Fox 2003).

**Figure 8:**
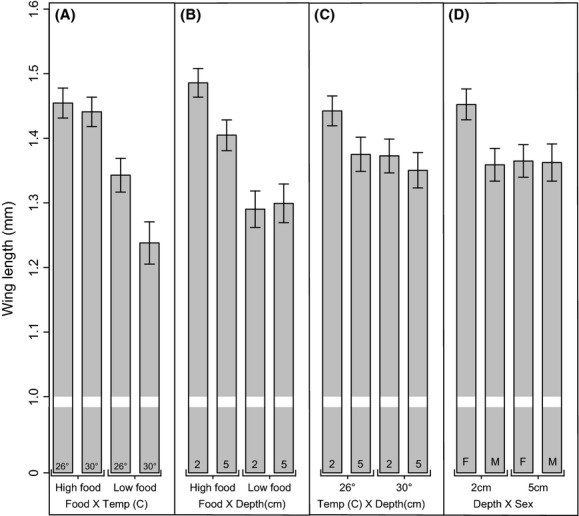
Adjusted mean wing length by groups showing interactions among factors. Panel A shows food-by-temperature, B shows food-by-depth, C shows temperature-by-depth, and D shows depth-by-sex. High- and low-food labels correspond to 0.2 and 1.4 mg per larva per day. Adjusted means and 95% Confidence intervals were calculated using the effects package in R (Fox 2003).

Wing length was affected by all three experimental factors as well as sex and the interactions food-by-temperature, food-by-depth, depth-by-temperature, and depth-by-sex (Table 2). More food increased wing length (Figs. 6 and 7A), deeper water decreased wing length (Figs. 6 and 7C), and higher temperature decreased wing length (Figs. 6 and 7B). Females generally had longer wings than males (Figs. 6 and 7D). For the food-by-temperature interaction, higher temperature produced a much greater decrease in wing length under low food than high (Fig. 8A). For the food-by-depth interaction, deeper water produced a much greater decrease in wing length under high food than low (Fig. 8B). For the temperature-by-depth interaction, deeper water caused greater reduction in wing length at lower temperature (Fig. 8C). And for the depth-by-sex interaction, females had longer wings than males in shallow water, whereas both sexes had equal wing lengths in deeper water (Fig. 8D).

**Figure 9:**
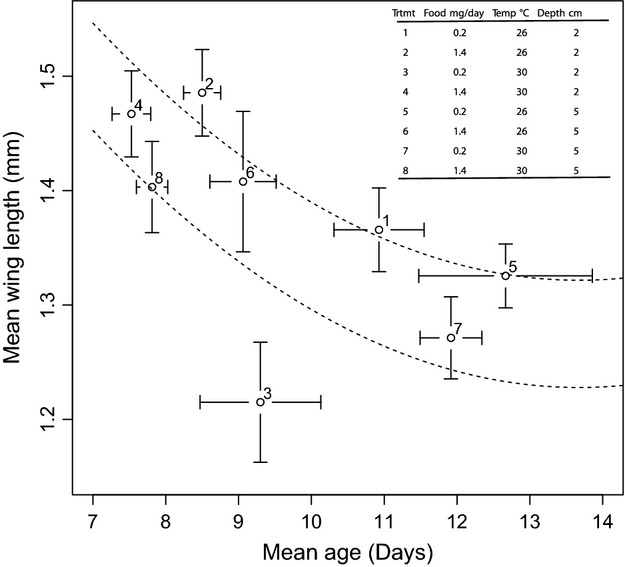
Bivariate means for age and size at emergence. Mean age at emergence and wing length of *Anopheles gambiae* sensu stricto in response to different food, water depth, and temperature levels. Confidence intervals are 95%. Treatment ID numbers are shown with corresponding factor levels. Dashed lines show a polynomial linear model with temperature as a covariate. Upper line shows 26°C and lower shows 30°C. Data are suggestive of two age–size reaction norms.

The response variables age at emergence and wing length are nonindependent, and the figure showing bivariate age–size means for each of the experimental treatments suggested a roughly L-shaped pattern (Fig. 9). Such a pattern is common across gradients of environmental qualities for this and other species (see Introduction). We conducted a polynomial regression of age at emergence and wing length to approximate an L-shaped age–size reaction norm. The model was statistically significant (adjusted *R*^2^ = 0.1907; *P* < 0.0001). Separate L-shapes appeared to be repeated within each of the two temperature levels, and we added temperature to the model to evaluate whether the data were better described using two curves. The resulting two-curve ANCOVA provided a substantially better fit (adjusted *R*^2^ = 0.3408; *F*_1,102_ = 24.462; *P* < 0.0001; Fig. 9).

## Discussion

In nature, multiple different environmental factors vary, potentially interacting in nonlinear and nonintuitive ways. Our factorial rearing experiment showed that different levels of food, water depth, and temperature produced interaction effects in age and size at emergence of the malaria vector mosquito *An. gambiae* s.s. (Tables 1 and 2; Figs. 5 and 8). These results demonstrate that how individual factors affect this species depends on the levels of other factors in the environment. This finding is consistent with other studies of mosquitoes and other insects (Colbo and Porter 1981; Lyimo et al. 1992; Leonard and Juliano 1995; Juliano 1996; Walker et al. 1997a; Baltzley et al. 1999; Agnew et al. 2002; Knight et al. 2004; Stillwell et al. 2007; Triggs and Knell 2011).

Population dynamics of insects are shaped heavily by both development time and adult size, and interactions affecting these life-history traits pose a challenge for modeling how populations of socially and economically important insects will respond to new environments. It is not feasible to experimentally assess life-history responses under all relevant climate and land-use scenarios; there are too many combinations of levels among too many factors. A more fruitful approach would be to identify and better understand the underlying causes of interactions. Such mechanistic insight would inform useful models of mosquito dynamics and malaria risk (Parham et al. 2012). How environmental conditions affect insect life histories is a consequence of how developing insects manage their growth and development. It is unlikely that mosquitoes and other insects maintain separate rules governing development for every possible set of conditions; more likely they respond to internal states that are determined by environmental conditions, physiology, and their evolved developmental programs. Thus we discuss our results in the context of energy budgets and the relationship between age and size.

Before proceeding, it is important to consider that the observed effects on age and size may have resulted from differential survival among treatment groups. There is good evidence for plastic development in *An. gambiae* s.s. and other mosquito species (Briegel 2003), and we generally interpret our results in the context of how larvae adaptively adjust development. However, survival was affected by food level, and both larval development time and body size show substantial heritability in *An. gambiae* s.s. (Lehmann et al. 2006). Thus, differential survival among phenotypes offers an alternate explanation of observed effects. However, if differential survival was wholly responsible for the observed treatment effects, the ranges of responses under low-food treatments would be subsets of the high-food treatments – and they are not. From a graphical inspection of the wing length data, the low end of the high-food distribution did not overlap with the low end of the low-food range. Similarly, the longest developmental durations under high food did not overlap with the extremes produced under low food. Furthermore, food level was the only factor found to significantly affect survival. Thus treatment effects can reasonably be attributed to plastic development.

Also, larvae reared individually in vials may poorly represent processes of natural systems. However, our experimental findings provide insight into how patterns of plastic growth and development can contribute to complex environmental effects.

### Separate environmental variables

Food has a relatively large effect on growth and development because it provides the matter that gets incorporated during growth and the energy used in biological processes. Of the three experimental factors, food produced the greatest effects on age at emergence, wing length, and survival (Figs. 4, 7, and 2). Larvae with more food developed faster and reached larger final sizes. These effects were clearly evident in the overall pattern of age and size at emergence; in Figure 9, the high-food treatment groups all had distinctly greater mean wing lengths and lower mean ages than the low-food treatments. These results were consistent with other studies (Timmermann and Briegel 1998; Gimnig et al. 2002). Furthermore, from C. Phelan unpubl. data, higher food availability can produce as much as a 250% increase in body weight and 50% reduction in development time of *An. gambiae* s.s. In the factorial experiment, food also contributed to four of the six significant interaction effects (Tables 1 and 2).

Deeper water slowed down development and reduced adult body size (Figs. 4C and 7C). This pattern is the inverse of increased food. Previous publications suggest that *An. gambiae* s.s. feed exclusively at the water surface (Kaufman et al. 2006; Klowden 2007). However, larvae in culture spend substantial time at the bottoms of their rearing containers where food accumulates, and they have been shown to ingest this food (A.G. Hoi, unpubl. data). Furthermore, Timmermann and Briegel (1993) found that rearing *An. gambiae* s.s. in water deeper than 2 cm increased mortality, and in a study of larval diving behavior of *An. gambiae* s.s., Tuno et al. (2004) similarly found greater mortality with deeper water. Animals that dive use up energy and oxygen getting to depth (Leeuw 1996; Tuno et al. 2007). The experimental effects of water depth on age and size at emergence may reflect an energetic cost of diving to forage.

With respect to temperature, larvae in warmer water emerged earlier and smaller (Figs. 4B and 7B). These temperature effects differed from the food and depth effects in that age and size at emergence both changed in the same direction (increased or decreased together). This special effect of temperature is common across taxa and is reflected in the temperature–size rule (Angilletta et al. 2004). For poikilothermic organisms, higher rearing temperatures both speed up development and increase metabolic rate (Kooijman 2009). Temperature's effect on developmental rate distinguishes it from other factors. Higher temperature, like less food, produces adults with smaller wings. However, high temperature also speeds up development to reduce age at emergence, an effect that is inconsistent with a simple reduction in available energy.

### Interactions among variables

Separately, the effects of each of the three experimental factors are straightforward, but statistical interactions show that development is context-dependent. Changes in a given environmental variable can have substantial effects at one level of a second variable but none, or opposite effects, at another. Furthermore, which life-history trait is affected, age or size at emergence, is similarly context-dependent.

Insight into some of the observed interaction effects is given by how the different factors affect larval energy usage and the nonindependent relationship between age and size. Under an L-shaped age–size reaction norm (see Introduction), more energy for growth generally means earlier emergence at a larger size. An important consequence of this L-shaped relationship is that when growth conditions are good (i.e., energy is abundant such that larvae end up in the vertical part of the L) small differences in conditions among environments will yield responses in adult size but not time to emergence, which is minimized. Conversely, if growth conditions are generally poor (the horizontal part of the L), small differences yield a response in time to emergence but not body size, which is minimized. In other words, for medium-to-good conditions age at emergence is fixed at some minimum value, whereas for medium-to-poor conditions size becomes fixed, again at a minimum. These properties of the age–size reaction norm offer explanations of the food-by-depth interactions observed for both age and size at emergence. These and other interaction effects are discussed below.

For age at emergence, there was a food-by-depth interaction (Fig. 5A). Under low food, larvae emerged substantially later when they were in deeper water. Possibly, depth only affected days to emergence under low food because, under the L-shaped reaction norm, age is fixed at a minimum when growth conditions are generally good (under high food). Alternately, the food-by-depth interaction could be explained by bottom foraging. Diving in deeper water may require more energy because more dives (or longer) are needed to get food from the bottom when it is less abundant: individuals must do more to get more (Boyd 1997). Possibly, under high food this extra activity yields enough resources to compensate for the extra cost of foraging, whereas under low food it does not.

A food-by-depth interaction was also present for wing length, but it followed an inverse pattern to that of age at emergence. A difference in wing lengths occurred between depths only under high food (Fig. 8B). Again, this is likely because the L-shaped age–size reaction norm allows flexibility in size (i.e., wing length) only under nutritionally rich conditions. The effect of water depth was expressed by wing length only under high food because the high-food treatments fall out along the vertical (size) dimension of the L-shaped age–size curve.

Thus, to get a full picture of how water depth effects mosquito development across food levels both age and size at emergence must be considered; depth affects age at emergence under high food and wing length under low food. In the context of the age–size reaction norm, food-by-depth interactions can be explained in terms of supply and usage of metabolic energy.

For age at emergence, there was also an interaction between food and sex (Fig. 5B). Under low-food females took substantially longer to develop than males. A simple explanation of this is that females are generally larger than males (Fig. 7D) and under low food it takes longer to achieve this size difference.

For wing length, there was an interaction between food and temperature (Fig. 8A). Wing length was much smaller at high temperature than low, but only under low food. Temperature speeds up developmental rate and increases food requirements such that it can be considered equivalent to a transformation of time (Kooijman 2009). Under high food, larvae may have had enough energy to become as large as possible despite greater energetic costs at higher temperature. Under low food, however, larvae at the higher temperature may have been forced to adjust their size to the limited available energy.

There was another wing length interaction between depth and temperature (Fig. 8C). Under low temperature wing length was greater at low depth, whereas there was no significant difference at high temperature. This pattern lacks an obvious explanation. Experimental treatment levels for temperature and water depth had very similar overall effects on wing length (Fig. 7B and C), and it remains unclear why low temperature would produce a larger difference in wing lengths between the two water depths (or vice versa). Possibly the increase in rate of development from the higher temperature limited time available for growth, and because growth is a nonlinear process a longer developmental time under low temperature allowed for relatively large gains in size at the more favorable shallow depth.

Finally, for wing length there was an interaction between water depth and sex (Fig. 8D). In shallow water only, there was a large difference in wing length between sexes. Females are larger than males (Fig. 7D) except, it seems, when both are reared in deep water. This result is also difficult to explain. If this were a consequence of simple differences in energy costs between living in deep and shallow water there should be a similar food-by-sex interaction, but there is not. Perhaps an effect of body size on oxygen budgets is at work: a higher cost of being underwater for larger larvae might produce such a result (Verberk and Bilton 2011).

### A temperature-dependent age–size reaction norm

Temperature appears to fundamentally differ from food and water depth in how it affects the L-shaped bivariate age–size reaction norm. In Figure 9, the positions of the four food-depth treatment level combinations are suggestive of two distinct L-shaped age–size curves within each temperature. We used polynomial regressions to test whether the data supported a model with separate curves for each temperature. A two-curve model (ANCOVA) explained the data substantially better than a single-curve one. If the age–size reaction norm was simple (Stillwell et al. 2007) – that is, if the slope or shape of the age–size curve were unaffected by changes across different variables – then a change in any given factor effectively adjusts a single universal meta-factor – “environmental quality” or net energy available for growth. A complex scenario, as these data for *An. gambiae* s.s. suggest, indicates context-dependence that needs to be unraveled to understand mosquito development under novel environments. Recall that only temperature caused age and size to both change in the same direction (e.g., lower temperature produced greater development time and larger size). Temperature's distinctive effects appear to extend to the age–size reaction norm. To explain L-shaped age–size reaction norms, Day and Rowe (2002) proposed a formal model with a minimum size threshold. Our results suggest that temperature adjusts the level of such a threshold. However, the eight treatment combinations from this study merely suggest such a pattern. This idea could be tested by rearing larvae across several food levels at two or more temperatures.

## Conclusions

Our factorial rearing experiment clearly demonstrates that environmental factors do not act independently of one another to affect important life-history traits of the malaria vector *An. gambiae* s.s. In mosquitoes and other insects, age and size at maturity often follow an L-shaped relationship across a range of growth conditions. This pattern offers an explanation for some of the interactions we observed. Moreover, temperature may have caused this age–size reaction norm to shift vertically. This ubiquitous age–size pattern may be useful for understanding complex environmental effects on life-history traits.

Dependencies among factors will determine how populations of insects respond to environmental change. Many insect species are economically and socially important, and further research is needed to reveal mechanisms that underlie such complex environmental effects. Because testing effects of multiple factors is logistically problematic, this issue is best approached from an organismal perspective, focusing on how distinct factors influence energy usage and rates of development within the context of an evolved developmental program.
